# Complications among Patients Undergoing Pancreaticoduodenectomy in Tertiary Care Centers of Nepal: A Descriptive Cross-sectional Study

**DOI:** 10.31729/jnma.7050

**Published:** 2022-01-31

**Authors:** Harish Chandra Neupane, Tseten Yonjen Tamang, Santosh Timalsina, Kishor Kumar Tamrakar, Abhishek Bhattarai

**Affiliations:** 1 Department of Surgery, Chitwan Medical College, Bharatpur-10, Nepal; 2 Research Unit, Chitwan Medical College, Bharatpur-10, Nepal

**Keywords:** *developing countries*, *pancreaticoduodenectomy*, *pancreatic fistula*

## Abstract

**Introduction:**

Pancreaticoduodenectomy (Whipple procedure), even after significant evolution, continues to be associated with a high morbidity. The study aimed to find out the prevalence of complications following pancreaticoduodenectomies performed by a single surgeon over a span of 20 years in tertiary care hospitals of Nepal.

**Methods:**

This was a descriptive cross-sectional study conducted from hospital records of patients who underwent pancreaticoduodenectomy between 1999 and 2019 at different institutions in Chitwan, where the principal author was involved. Ethical clearance was taken from the Institutional Review Committee. Convenience sampling was done. Patients' clinical characteristics and diagnoses were noted. Data entry was done using Statistical Package for the Social Sciences version 20. Point estimate at 95% Confidence Interval was calculated, with frequency and percentage.

**Results:**

Out of 327 patients who underwent pancreaticoduodenectomy, complications were found in 125 (38.2%) (32.9-43.7 at 95% Confidence Interval). Respiratory complications were the commonest 32 (9.8%), followed by septicemia 25 (7.6%) and cardiac complications 24 (7.3%). Delayed gastric emptying and postoperative pancreatic fistula were seen in 11 (6.8%) and 4 (2.5%) in the first decade respectively. In the second decade, delayed gastric emptying was noted in 2 (1.2%) and postoperative pancreatic fistula in 1 (0.6%) patient.

**Conclusions:**

The prevalence of complications in our study was comparable to other national and international studies. Surgery-specific complications such as delayed gastric emptying and postoperative pancreatic fistula showed a decline over the decade.

## INTRODUCTION

Pancreaticoduodenectomy (PD) is the procedure of choice for numerous benign and malignant tumours of the pancreas and periampullary region. The evolution of PD began with William Halstead operating on a duodenal tumor, and later the procedure was popularized by Allen Whipple.^1-3^ Since then, it has evolved not only by expansion of the indications but also in the surgical technique and the perioperative management.

PD is a technically demanding surgery with high morbidity and mortality. Pancreatic fistula, delayed gastric emptying (DGE) and pancreatic hemorrhage are dreaded post-operative complications and numerous mitigation strategies have been described.^2^ Studies also associate mortality and morbidity rates with the surgeons' experience; learning curve and volume of cases performed annually.^4,5^

The aim of this study was to find out the prevalence of complications following pancreaticoduodenectomy performed over a span of 20 years by a single surgeon at different tertiary care centers of Nepal.

## METHODS

This was a descriptive cross-sectional study that included hospital records of adult patients (>25 years) who underwent PD between 1999 and 2019. Ethical approval was taken from the Institutional Review Committee (IRC) of Chitwan Medical College (Ref: CMC-IRC/077/078-270). The study included clinical records of the eligible patients where the first author was the operating surgeon. Patients underwent PDs at three tertiary care health centers situated at Bharatpur, Chitwan viz. B.P Koirala Memorial Cancer Hospital, College of Medical Sciences, and Chitwan Medical College. Patients that had incomplete records were excluded from the study. Convenience sampling was done and the sample size was calculated as follows,

n = Z^2^ × (p × q) / e^2^

  = (1.96)^2^ × 0.58 × (1-0.58) / (0.06)^2^

  = 260

Where,

n = minimum required sample size,
Z= 1.96 at 95% Confidence Interval (CI),
p = prevalence of complications following pancreatoduodenectomy, 58%^2^
q = 1-p,
e = margin of error, 6%

Taking a 10% non-response rate, the sample size was 286. However, we included 327 patients in the study.

The patient data included demographics, clinical presentation, co-morbid factors, histopathological diagnosis, operative parameters [operative time, estimated blood loss (EBL)], post-operative parameters i.e., 30-day morbidity and mortality, and days of hospital stay.

All patients eligible for surgery were initially evaluated with a CECT abdomen (pancreas protocol) to assess resectability. All patients underwent routine preanesthetic evaluation, with echocardiography and pulmonary function test warranted in selected patients (e.g. age >45 years, presence of co-morbidities). There was a gradual transition (around 2003) of technique from non-pylorus preserving PD to pylorus-preserving PD in the second decade. All pancreato-enteric anastomosis was an end-to-end pancreatojejunostomy (PJ). The dunking method was preferred initially with a gradual transition to mucosa-to-mucosa PJ in the second decade. Similarly, the preference of suture material also changed from absorbable [polyglactin (vicryl)/silk] to non-absorbable [polypropylene (prolene)] for PJ over the last few years. Roux-en-Y gastrojejunostomy (GJ) was performed in all resections. Histopathological examination (HPE) was sent after the resection.

Post-operatively, patients were routinely kept under nasogastric (NG) drainage and NPO for the first 4 days. Ciprofloxacin, metronidazole, and gentamycin were used in the first decade as prophylactic antibiotics. Gradually, it was replaced by ceftriaxone along with metronidazole over the decade.

Data was entered into Statistical Package for the Social Sciences version 20. Descriptive statistics were performed and point estimate at 95% CI was calculated along with frequency and proportion for binary data and mean±SD for continuous data.

## RESULTS

Among the 327 patients undergoing PD, the prevalence of complications was 125 (38.2%) (32.9-43.7 at 95% CI). The most frequent complications were respiratory 32 (9.8%), septicaemia 25 (7.6%) and cardiac 24 (7.3%). DGE was observed in 13 (4%) whereas IAA, PPH and POPF each were seen in 5 (1.5%) patients. Overall, readmission had to be done for 5 (1.5%) patients. The reasons for readmission included nausea, vomiting, septicaemia and/or IAA. Only one (0.3%) patient required reoperation for PPH but succumbed to the bleeding (Table 1).

**Table 1:** Complications observed in the patients undergoing pancreatoduodenectomy (n = 327).

Variables	Overall n (%)	1^st^ decade (n= 162) n (%)	2^nd^ decade (n= 165) n (%)
Specific complications			
DGE^*^	13 (4.0)	11 (6.8)	2 (1.2)
Intra-abdominal abscess	5 (1.5)	2(1.2)	3 (1.8)
Gastrointestinal	14 (4.3)	5 (3.1)	9 (5.5)
Respiratory	32 (9.8)	14 (9.6)	18 (10.9)
Cardiac	24 (7.3)	12 (7.4)	12 (7.3)
Renal	14 (4.3)	6 (3.7)	8 (4.8)
Neurological	5 (1.5)	2 (1.2)	3 (1.8)
PPH^†^	5 (1.5)	4(2.5)	1 (0.6)
POPF^‡^	5 (1.5)	4(2.5)	1 (0.6)
Bile leak	-	-	-
SSI^§^	10 (3.1)	5 (3.1)	5 (3.0)
Septicemia	25 (7.6)	14 (10.2)	11 (7.3)
Others	22 (6.7)	11 (6.8)	11 (6.7)
Readmission	5 (1.5)	2 (1.2)	3 (1.8)
Re-operation	1 (0.3)	1 (0.6)	0 (0.0)
Mortality	10 (3.1)	8 (4.9)	2 (1.2)
Total	125 (38.2)	66 (40.7)	59 (35.8)

* DGE= Delayed Gastric Emptying

† PPH= Postpancreatectomy hemorrhage

‡ POPF= Postoperative pancreatic fistula

§ SSI= Surgical site infection

Overall, the perioperative mortality was found in 10 (3.1%) (Table 1). All of them had malignant lesions. The causes of mortality were septicemia 5 (1.5%), PPH 3 (1.8%) and myocardial infarction 2 (1.2%). There was a decline in mortality over the decade from 8 (4.9%) to 2 (1.2%).

Out of the total surgeries, 162 were performed in the first decade (1999-2008 AD) and 165 in the second (2009-2019 AD). The median age was 62 years with range of 27-82 years. There was a female preponderance 187 (57.2%) and jaundice 163 (49.8%) was the most common presenting symptom. Just over half of the patients 168 (51.3%) had one or more comorbidities. Hypertension was the commonest comorbidity 90 (27.5%) followed by abdominal pain (20.2%). The demographic parameters and comorbidity status were comparable between the decades (Table 2).

**Table 2:** Sociodemographic characteristics and operative parameters of patients.

Variables	Overall (n= 327) n (%)	1^st^ decade (n= 162) n (%)	2^nd^ decade (n= 165) n (%)
Socio-demographic data			
Sex			
Male	140 (42.8)	71 (43.8)	69 (41.8)
Female	187 (57.2)	91 (56.2)	96 (58.2)
Symptoms			
Jaundice	163 (49.8)	76 (46.9)	87 (52.7)
Loss of weight	37 (11.3)	24 (14.8)	13 (7.9)
Abdominal pain	66 (20.2)	33 (20.4)	33 (20.0)
Nausea and vomiting	37 (11.3)	18 (11.1)	19 (11.5)
Others	24 (7.3)	11 (6.8)	13 (7.9)
Pre-existing co-morbidity			
HTN^*^	90 (27.5)	47 (29.0)	43 (26.1)
IHD^†^	22 (6.7)	13 (8.0)	9 (5.5)
Dysrhythmia	12 (3.7)	7 (4.3)	5 (3.0)
CVA^‡^	9 (2.8)	3(1.9)	6 (3.6)
PVD^§^	3 (0.9)	2 (1.2)	1 (0.6)
COPDf^¶^/ Asthma	27 (8.3)	10 (6.2)	17 (10.3)
Diabetes mellitus	32 (9.8)	14 (8.6)	18 (10.9)
Renal disorders	3 (0.9)	2 (1.2)	1 (0.6)
Others	55 (16.8)	30 (18.5)	25 (15.2)
Blood transfusion			
Needed	129 (39.4)	68 (42.0)	61 (37.0)
**Blood loss (ml)**	**Overall Median (Range)**	**1^st^ Decade Median (Range)**	**2^nd^ Decade Median (Range)**
	300 (0-5500)	350 (0-5000)	300 (0-5500)
**Operative parameters**	**Overall**	**1^st^ Decade**	**2^nd^ Decade**
	**Mean±SD**	**Mean±SD**	**Mean±SD**
Operative time (min)	261.2±55.1	263.8±51.4	258.6±58.5
Length of post-operative hospital stay (days), mean±SD	15.6±4.7	15.8±4.7	15.4±4.6

* HTN= Hypertension

† IHD= Ischemic Heart Disease

‡ CVA= Cerebrovascular Accident

§ PVD= Peripheral Vascular Disease

¶ COPD= Chronic Obstructive Pulmonary Disease

†† DM= Diabetes Mellitus

‡‡ FFP=Fresh Frozen Plasma

The overall operative time, blood loss (BL), need for blood transfusion and the length of hospital stay remained relatively unchanged over the decades.

Malignant tumour 274 (83.8%) was the most frequent histopathological diagnosis after PD; the commonest being periampullary carcinoma 232 (70.9%) followed by distal cholangiocarcinoma 13 (4.0%) and lymphoma 11 (3.45%) (Table 3). Between the decades, the histopathological characteristics of the diagnosis were comparable.

**Table 3:** Histopathological diagnosis of the patients.

Pathological diagnosis	Overall (n = 327) n (%)	1^st^ decade (n= 162) n (%)	2^nd^ decade (n= 165) n (%)
Benign	53 (16.2)	23 (14.2)	30 (18.2)
Adenoma	37 (11.3)	20 (12.3)	17 (10.3)
Duodenal polyp	2 (0.6)	0 (0.0)	2 (1.2)
Ganglionic tumor	1 (0.3)	0 (0.0)	1 (0.6)
Neuroendocrine tumor	1 (0.3)	1 (0.7)	0(0.0)
Pancreatitis	11 (3.4)	2 (1.2)	9(5.5)
Unspecified	1 (0.3)	0(0.0)	1 (0.6)
Malignant	274 (83.8)	139 (85.8)	135 (81.8)
Periampullary carcinoma	232 (70.9)	122 (75.3)	110 (66.7)
Cholangi ocarcinoma	13 (4.0)	7 (4.3)	6 (3.6)
Cystic mucinous adenocarcinoma	4(1.2)	3(1.9)	1 (0.6)
Intestinal adenocarcinoma	10 (3.0)	0 (0.0)	10 (6.1)
Lymphoma	11 (3.4)	6 (3.7)	5 (3.0)
Neuroendocrine tumor	4(1.2)	1 (0.6)	3 (1.8)

## DISCUSSION

PD is a curative surgical procedure performed for numerous periampullary indications. In our study, it was most commonly done for malignant tumours, periampullary carcinoma being the frequent most. The final histopathology report seemed to vary from different institutions in Nepal; ampullary carcinoma (14.3-70.8%), distal cholangiocarcinoma (5.71-12.5%), carcinoma head of pancreas (4.2-11.4%) and duodenal carcinoma (4.2-20%).^2,6^ The number of PDs for pancreatic cancer could have been less in Nepal due to late presentation and unresectability.^2^ Our study is limited by the absence of exact number of individual items that constitute a PAC. In studies outside Nepal, PAC has been reported to constitute 57% of total PDs; with a diagnosis of pancreatic adenocarcinoma the commonest.^1^ The distribution of age, gender and presenting symptoms were similar to other studies elsewhere.^1,2,7^

The surgery is quite technically demanding; therefore, a wide array of perioperative complications are associated with it. Our complication rate of 38.2% corroborates with other studies, the reported rates ranging from 30 - 58%.^2,8-10^. Due to the retrospective nature of the data and limited clinical data availability, we could not assess the Clavien Dindo classification of surgical complications.^11^ It has to be noted that refinement in the definitions of complications of PD^8,12^ could affect comparability of our observations with those reporting recent data. The incidence of surgery-specific complications such as POPF, IAA, and DGE in our study were low compared to other centers including high volume centers.^2,8,10,13^ Literature reports numerous reasons for reoperation such as IAA, PPH, biliocutaneous fistula, wound dehiscence, tracheostomy and GJ revision among many others.^7^ We only had one reoperation for PPH to which he succumbed. Only five (1.5%) patients required readmission, which is less compared to 37% reported in other studies.^7^

A large number of our patients had comorbidities, cardiac comorbidities (e.g. hypertension, ischemic heart disease, dysrhythmia etc.) in particular, compared to other series from Nepal.^2^ This could be the reason why we had a high rate of medical complications (23%) and related deaths postoperatively, a finding also observed by DeOliveira, et al.^9^ Several preoperative risk factors for postoperative complications and inhospital death after PD have been described, notably age, sex, ischemic heart disease and the extent of the operative procedure.^14,15,16^

Our study had 10 perioperative mortalities (3.1%), and each one of them had one or more comorbidities and had malignant tumours. Our perioperative mortality is similar to other high volume centers, which report a mortality of <5%.^1,10^ Numerous studies have indicated an inverse relationship between mortality and surgeons' years of experience while others have found a positive relationship.^4^ The latter has been attributed to senior surgeons operating on higher risk patients or due to psychomotor decline. In our study, a decline in the prevalence of DGE and POPF as well as mortality rate was observed from first to second decade, indicating that surgeons' experience has a part to play in improving the postoperative outcome of patients after PD.

Note worthily, a number of studies have correlated higher case volume with lower complication rates, shorter post-operative length of stay and lower financial costs^15-17^, and therefore have suggested a policy of centralization for PDs. The same viewpoint has been supported by a recent meta-analysis that included different countries with disparate health care systems.^18^ However, the experience with low-to medium-volume centers in developing countries suggest that similar outcomes can be achieved if a dedicated HPB surgical team and a specialized HPB unit is in place.^2,19^ A study by Bhatti AB, et al. in Pakistan reported acceptable short-term and long-term outcomes for PD in a low-volume center in a resource-constrained setting.^19^ The outcomes have been particularly better, irrespective of the volume, within an academic health care system.^20^ The author believes that Nepal needs to establish a high volume PD center where all the GI surgeons of Nepal can refer the eligible cases for better outcomes. The center can also serve as a training center to all the aspiring surgeons to hone their skills related to PD.

With the improvement in the expertise (operative technique, patient selection and identification of associated risk factors) and care pathways (uniform definition of complications and intensive perioperative management, multidisciplinary approach) over the decade, the morbidities associated with PD have reduced. Adhering to these established protocols could not be over-emphasized, in order to generate favorable outcomes.

There were several limitations to this study. The database we used did not have all the important clinical data and the risk factors (incomplete data), and therefore bias could be present while calculating morbidity and mortality. The database is collection of a single surgeon's surgeries at different institutions in Chitwan, therefore might not be representative of hospitals in Nepal where PDs are performed. Another limitation of our study was that we were unable to follow 90-day mortalities in the patients. Studies have suggested 90-day mortality to be a more legitimate measure of surgery-related deaths in PD;^21^ therefore, our mortality rates have been under-estimated.

## CONCLUSIONS

The prevalence of complications in patients undergoing PD in our study was comparable to other national and international studies. A decline in surgery-specific complications such as delayed gastric emptying and postoperative pancreatic fistula as well as mortality rates was noted between the decades of surgery. The morbidities following PD have reduced over the decade with an improvement in surgical expertise and patient care pathways.
